# Hypertension resistant to antihypertensive agents commonly occurs with the progression of diabetic nephropathy in Japanese patients with type 2 diabetes mellitus: a prospective observational study

**DOI:** 10.1186/1471-2369-13-48

**Published:** 2012-06-27

**Authors:** Hiroyuki Ito, Mizuo Mifune, Mariko Abe, Koshiro Oshikiri, Shinichi Antoku, Yuichiro Takeuchi, Michiko Togane, Shigenori Ando, Emiko Tsugami

**Affiliations:** 1 Department of Diabetes, Metabolism and Kidney Disease, Edogawa Hospital, 2-24-18, Higashi-Koiwa, Edogawa, Tokyo, 133-0052, Japan; 2 Pharmaceutical Department, Edogawa Hospital, 2-24-18, Higashi-Koiwa, Edogawa, Tokyo, 133-0052, Japan

**Keywords:** Hypertension, Antihypertensive agents, Chronic kidney disease, CKD stage, Type 2 diabetes mellitus, KDIGO

## Abstract

**Background:**

We investigated 1) the frequency of hypertension in patients with type 2 diabetes graded by the new classification of chronic kidney disease (CKD) reported by the Kidney Disease: Improving Global Outcomes (KDIGO) and 2) the number of antihypertensive agents needed to achieve treatment goals using a prospective observational study.

**Methods:**

A population of 2018 patients with type 2 diabetes mellitus was recruited for the study. The CKD stage was classified according to the eGFR and the urinary albumin excretion levels.

**Results:**

Hypertension was found in 1420 (70%) of the patients, and the proportion of subjects showing a blood pressure < 130/80 mmHg was 31% at the baseline. Although the mean blood pressure was approximately 130/75 mmHg, the rate of patients with a blood pressure of < 130/80 mmHg became limited to 41-50% during the observation period. The number of antihypertensive agents required for treatment was significantly higher at the endpoint (2.0 ± 1.3) than at the baseline (1.6 ± 1.2). Furthermore, it increased with the progression of the CKD stage at both the baseline and the endpoint of the observation. However, the frequency of subjects who did not achieve the blood pressure target was found to increase in the group demonstrating the later stage of CKD.

**Conclusions:**

Hypertension resistant to antihypertensive agents was common in the patients with type 2 diabetes mellitus and increased with the progression of CKD. Although powerful combination therapy using antihypertensive agents is considered necessary for the strict control of blood pressure, this became difficult in individuals who were in advanced stages as graded based on the eGFR and the urinary albumin excretion levels.

## Background

It is well-known that hypertension is common in the patients with type 2 diabetes mellitus [1,2]. Hypertension is a major risk factor for the onset and progression of diabetic micro-a and macrovascular complications, as well as hyperglycemia [3,4]. Furthermore, the risk for cardiovascular events synergistically increases in the patients with both diabetes mellitus and hypertension [5]. The target blood pressure is recommended to be less than 130/80 mmHg in order to prevent diabetic vascular events [6,7]. Although it was reported that angiotensin-converting enzyme inhibitors (ACEIs) and angiotensin II receptor blockers (ARBs) have vascular protective effects, especially in the patients with diabetic nephropathy [8-13], it is often difficult to control blood pressure using single agents, and combination therapy was needed in many diabetic patients with hypertension [14].

The frequency of hypertension is elevated with the progression of renal damage in both diabetic and non-diabetic patients. In the present prospective observational study, we investigated 1) the frequency of hypertension in the patients with type 2 diabetes as graded by the new classification of chronic kidney disease (CKD) reported by the Kidney Disease: Improving Global Outcomes (KDIGO) [15] and 2) the number of antihypertensive agents needed to achieve the treatment goals.

## Methods

### Ethics statement

This study was conducted according to the principles expressed in the Declaration of Helsinki. The Ethics Committees of Edogawa Hospital approved the protocol of this study and waived the need for written informed consent because the data were analyzed anonymously for this observation study based on the data stored in the hospital database.

### Study population and methods

A population of 2018 patients diagnosed with type 2 diabetes mellitus who underwent consecutive evaluations, including blood pressure, urinalysis and determination of the serum creatinine levels in the Department of Diabetes, Metabolism and Kidney Disease of Edogawa Hospital, Tokyo, Japan between April 2008 and March 2011 was recruited for the study. Antihypertensive agents were essentially initiated when a systolic blood pressure (SBP) ≥ 130 mmHg and/or a diastolic blood pressure (DBP) ≥ 80 mmHg persisted after the lifestyle modification. The selection of antihypertensive agents was determined by each patient’s physician during the prospective observation period.

The blood pressure was measured twice with the subjects in the sitting position after a 5 minute rest. The lower value of the two measurements was used for the study. Hypertension was defined as a SBP ≥ 140 mmHg and/or a DBP ≥ 90 mmHg. The participants currently using antihypertensive medications were also classified as positive for hypertension. The target blood pressure is less than 130/80 mmHg according to the JNC7 [6] and the guidelines proposed by the European Society of Hypertension and of the European Society of Cardiology [8].

The patients were divided into four groups according to their blood pressure status. Categories 1, 2, 3 and 4 were defined to be the subjects showing 1) SBP < 130 mmHg and DBP < 80 mmHg, 2) SBP < 130 mmHg and DBP ≥ 80 mmHg, 3) SBP ≥ 130 mmHg and DBP < 80 mmHg, and 4) SBP ≥ 130 and DBP ≥ 80 mmHg, respectively. The doses and specific drug classes of antihypertensive agents for blood pressure control were dependent on the judgment of each patient’s physician.

The number of antihypertensive agents used was expressed as the sum of antihypertensive agents such as thiazide diuretics, loop diuretics, aldosterone antagonists, alpha blockers, beta blockers, calcium channel blockers (CCBs), ACEIs, ARBs, renin inhibitors, and centrally-acting adrenergic drugs being used.

The estimated glomerular filtration rate (eGFR) was calculated using the formula reported by Matsuo *et al.*[16]. This equation originated from the MDRD study group [17] arranged for Japanese individuals, and it is recommended by the Japanese Society of Nephrology: eGFR (mL/min/1.73 m^2^) = 194 × Scr^-1.094^ × Age^-0.287^ × 0.739 (if female).

The CKD stage was classified according to the eGFR and the urinary albumin excretion (UAE). The GFR stage was graded as: G1, eGFR ≥ 90 mL/min/1.73 m^2^; G2, 90 mL/min/1.73 m^2^ > eGFR ≥ 60 mL/min/1.73 m^2^; G3a, 60 mL/min/1.73 m^2^ > eGFR ≥ 45 mL/min/1.73 m^2^; G3b, 45 mL/min/1.73 m^2^ > eGFR ≥ 30 mL/min/1.73 m^2^; G4, 30 mL/min/1.73 m^2^ > eGFR ≥ 15 mL/min/1.73 m^2^; and G5, 15 mL/min/1.73 m^2^ > eGFR [15]. The UAE is presented as the albumin-to-creatinine ratio (ACR; mg/g creatinine). The albuminuria stage was graded according to an analysis of a spot urine sample as: A1 (normoalbuminuria), ACR < 30 mg/g creatinine; A2 (microalbuminuria), 30 ≤ ACR < 300 mg/g creatinine; or A3 (macroalbuminuria), ACR ≥ 300 mg/g creatinine (or dipstick urinalysis revealing 2+, 3+ or 4+).

The HbA1c levels were determined by a high performance liquid chromatography method using an automated HLC-723 G7 analyzer (Tosoh Corporation, Tokyo, Japan) and calibrated by the Japan Diabetes Society (JDS) standard calibrators. The value for HbA1c (%) was estimated as a National Glycohemoglobin Standardization Program (NGSP) equivalent value (%) calculated by the formula: HbA1c = HbA1c (JDS) + 0.4, considering the relational expression of HbA1c (JDS) measured by the previous Japanese standard substance and the measurement methods and HbA1c (NGSP) [18].

### Statistical methods

All data are shown as the means ± SD. An analysis of variance (ANOVA) and the *χ*2 test were used for between-group comparisons of the continuous and categorical variables, respectively. A paired-*t* test was performed to determine the significance of the change in the number of antihypertensive agents. Differences of *P* < 0.05 (two-tailed) were considered to be statistically significant. The statistical software package JMP, version 8.0 (SAS Institute, Cary, NC, USA), was used to perform all of the analyses.

## Results

Table 1 shows the baseline clinical characteristics and the laboratory parameters of the patients. Hypertension was found in 1420 (70%) patients, and 31% of the subjects showed a blood pressure < 130/80 mmHg. No antihypertensive agent was administered to 259 patients with hypertension. The number of antihypertensive agents used was 1, 2, 3, 4, 5, 6 and 7 in 471, 403, 196, 63, 21, 6 and 1 patients, respectively. The mean number of antihypertensive agents being used by the patients with hypertension and diabetes was 1.6.

**Table 1:** The baseline clinical characteristics of the patients

	**%/Mean±SD**	**Number estimated (%)**
Age (years)	63 ± 12	2018 (100)
Men	62	2018 (100)
Duration of diabetes mellitus (years)	9.4 ± 9.8	1723 (85)
Current plus past smoking	59	1585 (79)
Drinkers ^#^	42	1671 (83)
Treatment for diabetes mellitus		2018 (100)
Diet only/OHA/insulin/GLP-1 analogue	17/56/27/0	
Body mass index (kg/m^2^)	24.9 ± 4.3	1995 (99)
Obesity ^##^	44	1995 (99)
Systolic blood pressure (mmHg)	139 ± 21	2018 (100)
Diastolic blood pressure (mmHg)	80 ± 14	2018 (100)
Hypertension	70	2018 (100)
Number of antihypertensive agents	1.6 ± 1.2	1420 (70)
Control of blood pressure		2018 (100)
Category 1	31	629
Category 2	4	90
Category 3	20	407
Category 4	44	892
HbA1c (%)	8.5 ± 2.2	1887 (94)
Glycoalbumin (%)	24.7 ± 8.4	206 (10)
Estimated GFR (mL/min/1.73 m^2^)	55 ± 20	2018 (100)

*OHA*: oral hypoglycemic agents, GFR: glomerular filtration rate.

^#^ Drinkers were defined as those who consumed more than 20 g/day of ethanol.

^##^ Obesity was considered to be present in individuals with a body mass index ≥ 25 kg/m^2^.

**Figure 1:**
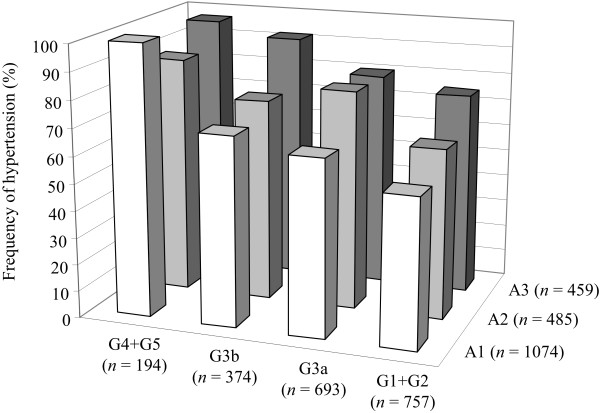
The frequency of hypertension in the groups subdivided by albuminuria (A1-A3) and GFR (G1+2, G3a, G3b and G4+5) stages.

The distribution of the patients divided by their albuminuria stage and GFR stage is shown in Table 2. The frequency of hypertension was significantly increased with the progression of the albuminuria stage (*P* < 0.01) and the GFR stage (*P* < 0.01) (Figure 1).

**Table 2:** The number of antihypertensive agents used in the patients with hypertension and diabetes at the baseline and at the endpoint of the observation

	**Albuminuria stage**							
	**A1**		**A2**		**A3**		**Average**	
**GFR stage**	**Baseline**^**$**^	**Endpoint**^**$**^	**Baseline**^**$**^	**Endpoint**^**$**^	**Baseline**^**$**^	**Endpoint**^**$**^	**Baseline**^**$**^	**Endpoint**^**$**^
G1 + G2	1.3 ± 1.0	1.7 ± 1.0	1.1 ± 1.0	1.7 ± 1.0	1.2 ± 0.8	2.0 ± 0.9^$§^	1.2 ± 1.0	1.7 ± 1.0
*n* (% of all)	261 (18.3)	182 (18.4)	127 (8.9)	79 (8.0)	58 (4.1)	37 (3.7)	446 (31.4)	298 (40)
G3a^#^	1.4 ± 1.0	1.7 ± 1.0	1.4 ± 1.1	1.8 ± 1.2	1.5 ± 0.9	2.3 ± 1.2	1.4 ± 1.0	1.8 ± 1.1
*n* (% of all)	269 (18.9)	217 (21.9)	140 (9.9)	82 (8.3)	86 (6.1)	68 (6.9)	495 (34.9)	367 (37.1)
G3b^#^	1.9 ± 1.1	2.1 ± 1.2	1.5 ± 1.3	2.0 ± 1.1	2.0 ± 1.3	2.6 ± 1.4	1.9 ± 1.3	2.3 ± 1.3^*^
*n* (% of all)	123 (8.7)	93 (9.4)	63 (4.4)	40 (4.0)	106 (7.5)	79 (8.0)	292 (20.6)	212 (21.4)
G4 + G5	3.0 ± 1.6	3.2 ± 1.8	2.4 ± 1.6	2.6 ± 1.2	2.5 ± 1.3	2.9 ± 1.4	2.5 ± 1.4	2.9 ± 1.4
*n* (% of all)	10 (1.0)	6 (0.9)	22 (1.5)	8 (0.8)	155 (10.9)	99 (10)	187 (13.2)	113 (11.4)
Average^*#^	1.5 ± 1.1	1.8 ± 1.1	1.4 ± 1.2	1.8 ± 1.1	2.0 ± 1.3	2.5 ± 1.4	1.6 ± 1.2	2.0 ± 1.3
*n* (% of all)	663 (46.7)	498 (50.3)	352 (24.8)	209 (21.1)	405 (28.5)	283 (28.6)	1420 (100)	990 (100)

^*^P < 0.01 among A1, A2 and A3 at baseline and ^#^*P* < 0.01 among A1, A2 and A3 at endpoint.

^$^*P* < 0.01 among G1 + G2, G2, G3 and G4 + G5.

**Figure 2:**
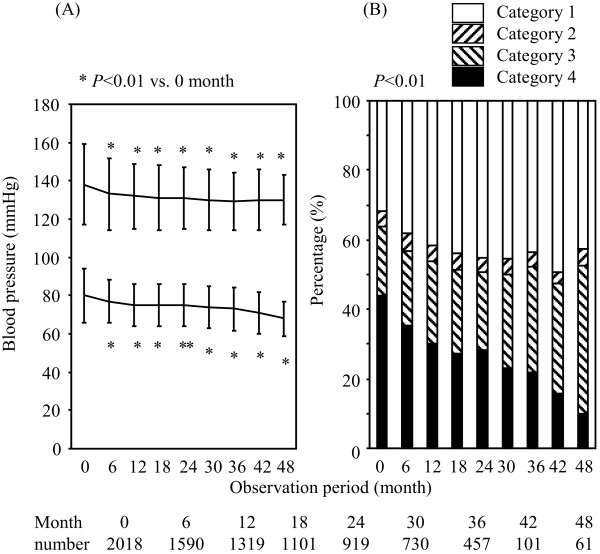
**(A) The blood pressure and (B) status of blood pressure control during the follow-up period.** The data represent the means ± SD.

**Figure 3:**
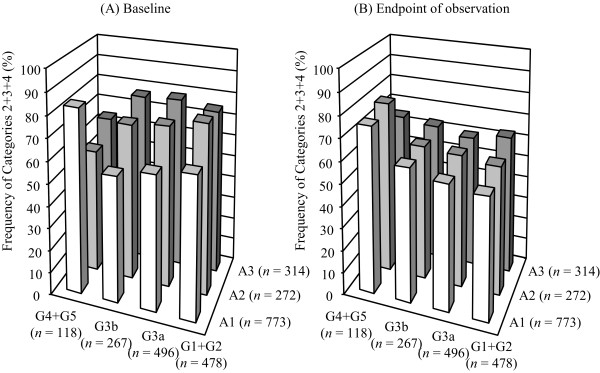
The percentages of individuals who did not achieve the target blood pressure (Categories 2+3+4) among the patients with different CKD stages at the baseline (A) and at the endpoint of observation (B) in the total of 1359 patients who were observed for more than 12 months.

Figure 2-A shows the blood pressure during the observation period in all subjects. Although the mean blood pressure was improved (132/75 mmHg, 131/75 mmHg, 129/73 mmHg and 130/68 mmHg at 12, 24, 26 and 48 months, respectively), the rate of patients who were in category 1 (SBP < 130 mmHg and DBP < 80 mmHg) was limited to 41-50% of the patients (Figure 2-B). In the 1359 patients who were observed for more than 12 months, the final blood pressure was 131 ± 16/74 ± 12 mmHg, and the rate of patients in category 1 was 43%. The percentages of individuals who did not achieve the target blood pressure (Categories 2 + 3 + 4) were decreased at the endpoint of the observation compared with the baseline (Figure 3). They percentage increased in the subjects demonstrating later stage of the albuminuria (*P* < 0.01) and the GFR (*P* < 0.01) at the endpoint of the observation (Figure 3-B).

Table 2 shows a comparison of the number of antihypertensive agents used in the patients with hypertension and diabetes at the baseline (*n* = 1420) and at the endpoint of the observation for more than 12 months (*n* = 990). The number of antihypertensive agents was significantly higher at the endpoint than at the baseline. Furthermore, it increased with the progression of CKD stage at both the baseline and the endpoint of the observation period.

CCBs, ACEIs, ARBs and thiazide diuretics were used by 52%, 15%, 55% and 13% of the patients at the baseline, and by 64%, 23%, 65% and 7% at the endpoint of the observation period.

## Discussion

In the present study, a much higher frequency of hypertension was found in the patients with type 2 diabetes mellitus. It could be argued that this is because our study included a number of CKD patients, although our results were consist with previous reports that showed hypertension to be frequently complicated with diabetes [1,2]. Our investigation is the first report showing that the frequency of hypertension was elevated with the progression of CKD, and that more drugs were needed for the treatment of hypertension during the progression of CKD. Furthermore, the percentage of patients who achieved the blood pressure target decreased in those demonstrating the later stage of CKD, despite their increased use of antihypertensive agents.

Bakris et al. reviewed the various clinical trials for either diabetes or renal impairment. An average of 3.2 different antihypertensive medications was taken daily in the UKPDS [3], ABCD [19], MDRD [20], HOT [21] and AASK [22] trials, even though the average blood pressure in these patients was slightly less than 140/90 mmHg [14]. The mean blood pressure of our study reached approximately 130/75 mmHg at the endpoint of observation using fewer drugs. The differences were considered to be caused by the use of more recent antihypertensive agents, such as long-acting CCBs and ARBs. Because the essential antihypertensive agents were thiazide diuretics, beta blockers, CCBs and/or ACEIs in these previous investigations, it is difficult to make a simple comparison to the present study.

The target value of blood pressure has continued to decline after the JNC I [23] was published as the first guideline for the treatment of hypertension in 1977. As strict control is required, especially in diabetic patients, we also tried to keep the blood pressure less than 130/80 mmHg in the present study. However, our current treatment protocol was considered to be insufficient for blood pressure control in patients with type 2 diabetes, even when a combination using two or more antihypertensive drugs was administered (the subjects who met the treatment goal comprised only ~50% of the population at the endpoint). Furthermore, tight control became more difficult as the CKD stage progressed. Unlike previous clinical studies, we are currently able to use various antihypertensive agents showing more powerful effects such as can be achieved using long-acting CCBs, ACEIs, ARBs and renin inhibitors. More aggressive treatment strategies, including some combination tablets, are therefore desirable in addition to the lifestyle modifications, in order to increase the likelihood that the target blood pressure can be reached and maintained.

Therapeutic resistance to antihypertensive agents in the late stage of CKD with diabetes mellitus is considered to be caused some defective mechanisms of vascular homeostasis and impaired nitric oxide production [24]. Vascular repair, according to a renal hemodynamic study [25], and nitric oxide production from the renal endothelial cells [26], have both been reported to be adequately functional in patients with the early stage of diabetic nephropathy. However, the number of antihypertensive agents had been higher during the entire observation period, even in the early stage (G1 + G2 and A1) of diabetic nephropathy, in the present study. This is thought to be due to the insufficient number of antihypertensive agents (1.3 ± 1.0) taken at the baseline by the G1 + G2/A1 stage patients.

The present study has several important limitations. First, the follow-up period was relatively short, and the number of patients decreased to nearly one-half at 24 months and to one-fourth at 36 months. This might have led us to incorrectly estimate the blood pressure control and the number of antihypertensive agents required for control. Second, we did not determine the dose of antihypertensive agents required. The doses administered were dependent on the judgment of each patient’s physician, as was the decision to add another antihypertensive agent or increase the dose of a drug that was already being used. When the dose increases, the enhanced antihypertensive therapy is not reflected in the number of antihypertensive agents. Third, the blood pressure measured at home was not evaluated in this study. If self-monitoring of blood pressure shows good control for hypertension, then physicians will not increase the therapy, even if the office blood pressure does not meet the target goal levels. This is considered to be one of the reasons why more than half of the patients remained within categories 2–4 in the present study. Fourth, the lower value of two measurements of blood pressure was used for this study, not the average value, which is recommended by the National Institutes of Health [6] and the Japanese Society of Hypertension [27]. This might have led to the underestimation of the blood pressure level observed in this investigation. Fifth, the number of patients gradually decreased during the observation period. It was possible that the omission of individuals with higher blood pressure had thus caused the improvement in the blood pressure that was seen during the observation period.

## Conclusion

In conclusion, hypertension was common in patient with type 2 diabetes mellitus and increased with the progression of CKD. Although powerful combination therapy using antihypertensive agents is considered to be necessary for the strict control of blood pressure, it becomes more difficult for individuals in the progressive stages of CKD as determined based on the albuminuria and GFR levels.

## Competing interests

The author(s) declare that they have no competing interests.

## Authors’ contributions

HI contributed to the conception and design of the study, research, analysis and interpretation of data, and the drafting of the manuscript. MM, MA, KO, SA, YT, MT, SA and ET contributed to the research and interpretation of the data, and to the critical revision of the manuscript. All authors have given their final approval of the submitted version of the manuscript. All authors read and approved the final manuscript.

## Pre-publication history

The pre-publication history for this paper can be accessed here:

http://www.biomedcentral.com/1471-2369/13/48/prepub
